# Expression of Placental Lipid Transporters in Pregnancies Complicated by Gestational and Type 1 Diabetes Mellitus

**DOI:** 10.3390/ijms25063559

**Published:** 2024-03-21

**Authors:** Paweł Jan Stanirowski, Mateusz Wątroba, Michał Pyzlak, Jarosław Wejman, Dariusz Szukiewicz

**Affiliations:** 1 1st Department of Obstetrics and Gynecology, Medical University of Warsaw, 02-015 Warsaw, Poland; 2 Department of Biophysics, Physiology and Pathophysiology, Faculty of Health Sciences, Medical University of Warsaw, 02-004 Warsaw, Poland; 3 Department of Pathology, Maria Sklodowska-Curie National Research Institute of Oncology, 02-781 Warsaw, Poland; 4 Department of Pathology, Professor Witold Orlowski Public Clinical Hospital, Medical Center of Postgraduate Education, 00-416 Warsaw, Poland

**Keywords:** placenta, lipoprotein lipase, endothelial lipase, fatty acid transport protein, fatty acid translocase, fatty acid binding protein, gestational diabetes mellitus, type 1 diabetes mellitus, fetal macrosomia

## Abstract

The objective of the study was to assess the expression of proteins responsible for placental lipid transport in term pregnancies complicated by well-controlled gestational (GDM) and type 1 diabetes mellitus (PGDM). A total of 80 placental samples were obtained from patients diagnosed with PGDM (*n* = 20), GDM treated with diet (GDMG1, *n* = 20), GDM treated with diet and insulin (GDMG2, *n* = 20), and a non-diabetic control group (*n* = 20). Umbilical and uterine artery blood flows were assessed by means of ultrasound in the period prior to delivery and computer-assisted quantitative morphometry of immunostained placental sections was performed to determine the expression of selected proteins. The morphometric analysis performed for the vascular density-matched placental samples demonstrated a significant increase in the expression of fatty acid translocase (CD36), fatty acid binding proteins (FABP1, FABP4 and FABP5), as well as a decrease in the expression of endothelial lipase (EL) and fatty acid transport protein (FATP4) in the PGDM-complicated pregnancies as compared to the GDMG1 and control groups (*p* < 0.05). No significant differences with regard to the placental expression of lipoprotein lipase (LPL) and FATP6 protein between GDM/PGDM and non-diabetic patients were noted. Maternal pre-pregnancy weight, body mass index, placental weight as well as the expression of LPL and FABP4 were selected by the linear regression model as the strongest contributors to the fetal birth weight. To conclude, in placentas derived from pregnancies complicated by well-controlled PGDM, the expression of several lipid transporters, including EL, CD36, FATP4, FABP1, FABP4 and FABP5, is altered. Nonetheless, only LPL and FABP4 were significant predictors of the fetal birth weight.

## 1. Introduction

**Figure 1:**
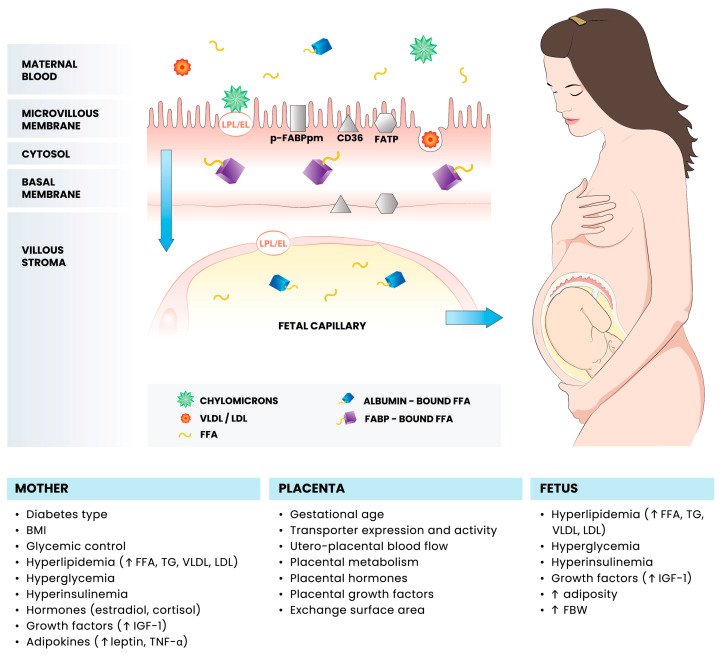
Schematic diagram of the maternal–fetal free fatty acid transfer in pregnancies complicated by diabetes mellitus. BMI, body mass index; CD36; fatty acid translocase; EL, endothelial lipase; FABP, fatty acid binding protein; FATP, fatty acid transport protein; FBW, fetal birth weight; FFA, free fatty acids; p-FABPpm, placental plasma membrane FABP; IGF-1, insulin-like growth factor 1; LDL, low-density lipoprotein; LPL, lipoprotein lipase; TG, triglycerides; TNFα, tumor necrosis factor alpha; VLDL, very-low-density lipoprotein.

The efficient supply of nutrients from the maternal circulation is of paramount importance for proper fetal growth and development. The pivotal role in the above-mentioned process is played by the placenta, which is primarily responsible for the regulation of the maternal–fetal flux of the amino acids, glucose and lipids. Among the numerous factors determining the placental capacity to transfer nutrients, such as utero–placental blood flow, concentration gradient or placental metabolism, the expression and activity of specific transporter proteins appears to be of particular importance. During pregnancy, lipids constitute an important group of nutrients, acting as cell membrane components and energy sources, thus being responsible for the adequate growth of the fetus and development of the fetal central nervous system [1]. Despite the relative solubility of lipids in plasma membranes, triglycerides (TG) contained in the maternal blood lipoproteins (chylomicrons, VLDL, LDL) are unable to cross the placental barrier and must first be hydrolyzed to free fatty acids (FFA) or endocytosed as intact lipoprotein particles (Figure 1) [1,2]. The hydrolysis process is initiated by the enzymes present in the microvillous membrane (MVM) of the syncytium, such as lipoprotein (LPL) and endothelial lipase (EL). Once cleaved from the TG, the FFAs penetrate the cell and are bound by facilitative transporters present in the membranes and cytosol of the trophoblast, including fatty acid translocase (CD36), fatty acid transport proteins (FATP) and fatty acid binding proteins (FABP) before being released into placental capillaries and fetal circulation. According to some of the hypotheses, the alterations in the placental expression of enzymes and proteins involved in the transport of FFA may be responsible for the excessive fetal fat accretion, and thus lead to disturbances of the fetal growth, such as fetal macrosomia [1,3].

Diabetes in pregnancy is considered as one of the main risk factors for fetal macrosomia [4]. According to the available data, up to 15.7% and 43.7% of pregnancies with concomitant gestational (GDM) or type 1 diabetes mellitus (PGDM) are affected by fetal overgrowth, respectively [5]. As a direct consequence, there is an increased rate of cesarean sections, birth trauma and neonatal mortality in pregnancies affected by GDM/PGDM [5]. Despite the underlying mechanisms being largely unknown, anthropometric studies conducted of fetuses and neonates from pregnancies complicated by GDM/PGDM have shown increased thicknesses of several skinfolds as well as the percentage of fat mass compared to normoglycemic gestations [6,7]. The above, combined with the increased FFA, TG and lipoprotein concentrations in the maternal serum and venous cord blood in GDM/PGDM women raises the question of possible disturbances in the transplacental lipid transfer under the conditions of a diabetic environment [8,9,10,11,12,13]. Indeed, several genes related to lipid transport encoding EL, FABP4 and FABP5 are upregulated in the placentas of GDM/PGDM women and multiple studies have demonstrated alterations in the expression of the enzymes and proteins responsible for the uptake of FFA in a diabetic pregnancy [14,15,16,17,18]. For example, the activity of LPL in the MVM was significantly increased among PGDM women in conjunction with the higher expression of FABP1 in GDM/PGDM populations [15]. Similarly, in the placental tissue derived from obese-GDM or PGDM women with sub-optimal glycemic control, the expression of EL mRNA was markedly elevated [16,17]. In contrast, reports on lean GDM patients showed significantly decreased EL and FATP4 mRNA levels, together with the enhanced expression of CD36 and FATP6 [18]. Finally, the results of several studies demonstrated an increased concentration of TG in the placentas of diabetic mothers, and there is some evidence of the stimulatory effect of insulin and FFA on the expression of proteins involved in lipid transfer across the placenta [11,17,19,20]. The above-mentioned observations allow us to hypothesize that the combined effects of hyperglycemia, hyperinsulinemia and dyslipidemia in patients with pregnancies complicated by diabetes mellitus lead to alterations in the expression and activity of proteins facilitating maternal–fetal transfer of FFA, increased placental lipid accumulation and ultimately to hyperlipidemia, increased adiposity and birth weight of the exposed fetuses [1,21].

Unfortunately, the results of the previously published studies performed in diabetic populations are inconclusive, and thus hinder testing of the proposed hypothesis. The main reasons for the observed discrepancies include small numbers of participants, different diagnostic criteria of GDM, lack of information concerning modes of treatment and glycemic control as well as combined analysis of GDM patients treated solely with diet with those requiring insulin therapy [11,15,16,18,20,22,23]. Finally, in numerous studies, whole placental homogenates were used for the evaluation of protein expression without a separate analysis of the trophoblast layer considered as the primary barrier for placental nutrient transport [11,15,20,22,24].

In the present study, we aimed to quantitatively evaluate the placental expression of proteins involved in the transfer of lipids in term pregnancies with concomitant well-controlled GDM and PGDM. In addition, based on the obtained results, independent contributors to the fetal birth weight (FBW) were assessed.

## 2. Results

Of the 60 diabetic patients that qualified for the study, 40 were diagnosed with GDM whereas 20 had pre-existing type 1 diabetes mellitus. The control group comprised an additional 20 normoglycemic women. Half of the patients with GDM required insulin therapy during the course of pregnancy. The demographic and clinical data of the study participants are shown in Table 1. In pregnancies complicated by PGDM, the median gestational age was significantly lower compared to the GDM and control groups (*p* < 0.001). No significant differences between the groups with respect to maternal pre-pregnancy weight were observed; however, the BMI in the GDMG2 and PGDM groups was higher when compared to the control group (GDMG2 25.2 kg/m^2^; PGDM 25.7 kg/m^2^ vs. control 21.4 kg/m^2^, *p* < 0.05). Additionally, patients with PGDM gained significantly more weight during pregnancy compared to both GDM groups (PGDM 15.5 kg vs. GDMG1 8.5 kg; GDMG2 11 kg, *p* < 0.05). Of importance, there were no significant differences between groups with regard to the percentage of women with a pre-pregnancy BMI exceeding 30 kg/m^2^. Fasting plasma glucose as well as 1 h and 2 h plasma glucose concentrations during OGTT were significantly increased among participants diagnosed with GDM as compared to the non-diabetic controls (*p* < 0.01). Moreover, a significant difference with regard to fasting plasma glucose concentration between GDMG1 and GDMG2 groups was noted (GDMG1 91 mg/dL vs. GDMG2 98.8 mg/dL, *p* < 0.01). Median third trimester HbA1c concentration among women with pre-existing diabetes was significantly higher when compared to the GDMG1 and GDMG2 groups (PGDM 5.9% vs. GDMG1 5.1%; GDMG2 5.4%, *p* < 0.001). Furthermore, both FBW and placental weights were significantly increased among women with PGDM in comparison with GDM and control groups (*p* < 0.05). The percentage of newborns weighing ≥4000 g reached 40% in the PGDM group and was higher than the ratio observed in the GDM and control groups (GDMG1 10%; GDMG2 10%; control 15%); however, the difference was not significant (*p* = 0.08). Ultrasound evaluation of the UA PI and UtA PI revealed no significant differences between groups, thus reflecting lack of major disturbances in the maternal–placental–fetal blood flow.

**Figure 2:**
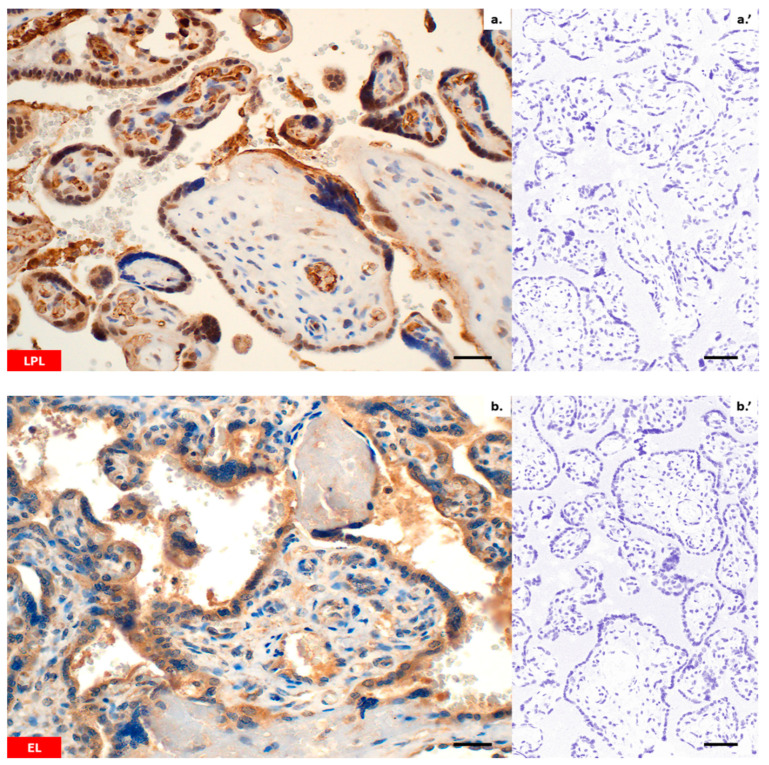

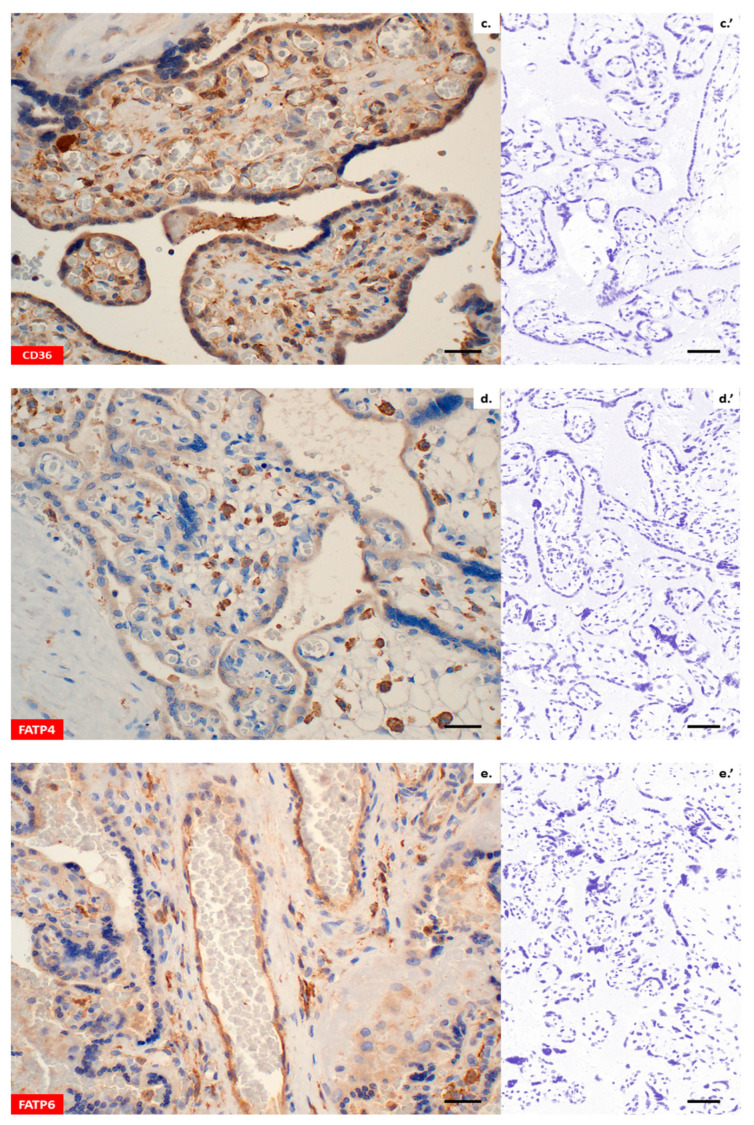

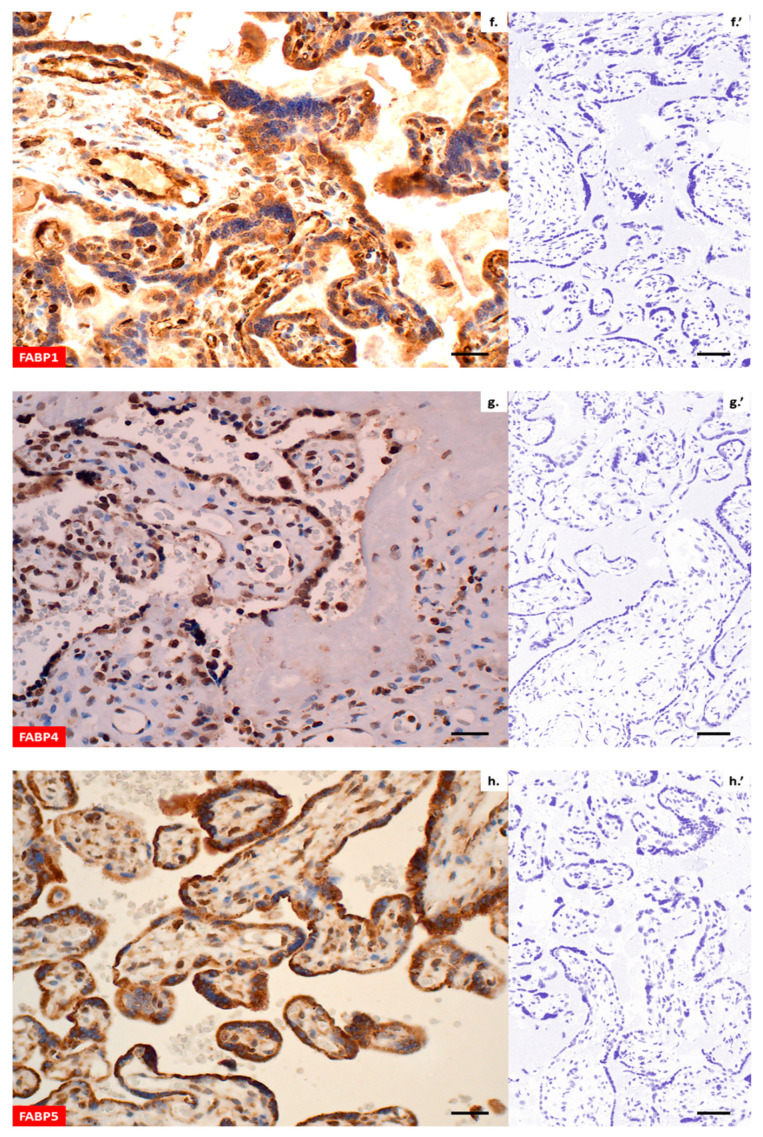
Immunohistochemical localization of proteins involved in the transport of lipids in human term placenta: LPL (**a**); EL (**b**); CD36 (**c**); FATP4 (**d**); FATP6 (**e**); FABP1 (**f**); FABP4 (**g**); FABP5 (**h**). Images (**a’**–**h’**) represent respective negative controls. Photomicrographs were captured at 400×. Scale bars = 50 μm.

Immunohistochemistry confirmed the presence of all lipid transporters in the placental sections (Figure 2a–h). Both, LPL and EL were found in the placental syncytiotrophoblast (ST), cytotrophoblast (CT) and vascular endothelium (VE) (Figure 2a,b). Likewise, immunohistochemical staining using CD36 antibodies revealed positive reactions in the membranes and cytosol of the ST, CT and VE (Figure 2c). With regard to FATP4 and FATP6, a diffuse cytoplasmic signal was found in the ST/CT or ST/CT and VE, respectively (Figure 2d,e). Moreover, both proteins displayed strong immunoreactivity in the Hofbauer cells of villous stroma (Figure 2d,e). Similarly to FATP4 and FATP6, the expression of FABP1, FABP4 and FABP5 was predominantly intracellular (Figure 2f–h). All proteins were present in the placental ST/CT. In addition, FABP1 was detected in VE (Figure 2f) and FABP4 in Hofbauer cells (Figure 2g). The negative control with rabbit pre-immune antibody demonstrated none of the immunoreactions described above (Figure 2a’–h’).

**Figure 3:**
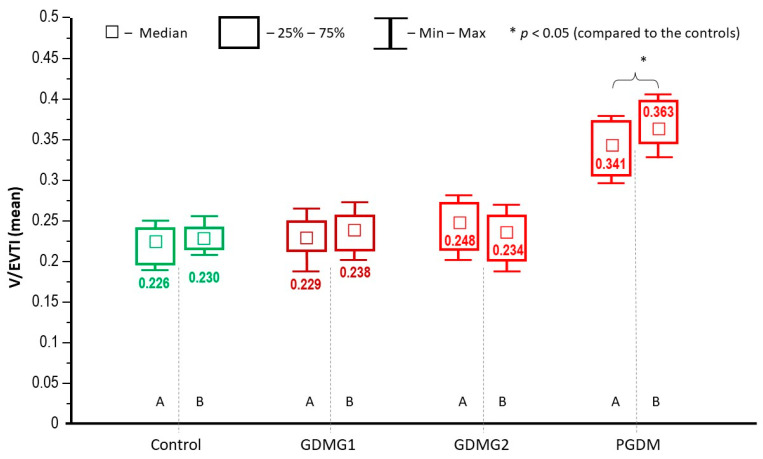
Analysis of the vascular/extravascular tissular index (abstract numbers and IQR) in the collected placental sections (A—central part, B—peripheral part of the placenta). GDMG1—diet-controlled gestational diabetes mellitus; GDMG2—insulin-controlled gestational diabetes mellitus; PGDM—type 1 diabetes mellitus.

A comparative analysis of the placental microvasculature revealed no significant differences with respect to median V/EVTI values between the central and peripheral placental specimens in any of the subgroups of patients (Figure 3). Hence, further analysis of protein expression was performed altogether for both placental specimens. Among women with PGDM, the median V/EVTI values from both placental regions were significantly higher as compared to other groups (*p* < 0.05) (Figure 3).

**Figure 4:**
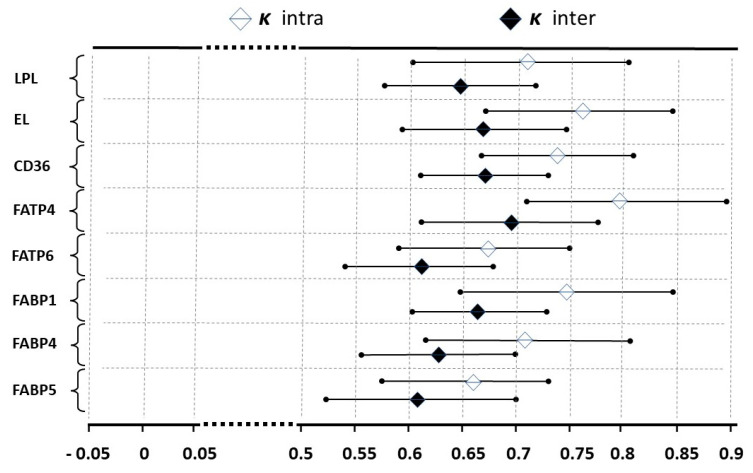
Results of the kappa statistic (*ĸ*) presenting intra- and inter-observer agreement with 95% confidence interval. The agreement is considered good (substantial to almost perfect) when the *ĸ* value is above 0.61.

**Figure 5:**
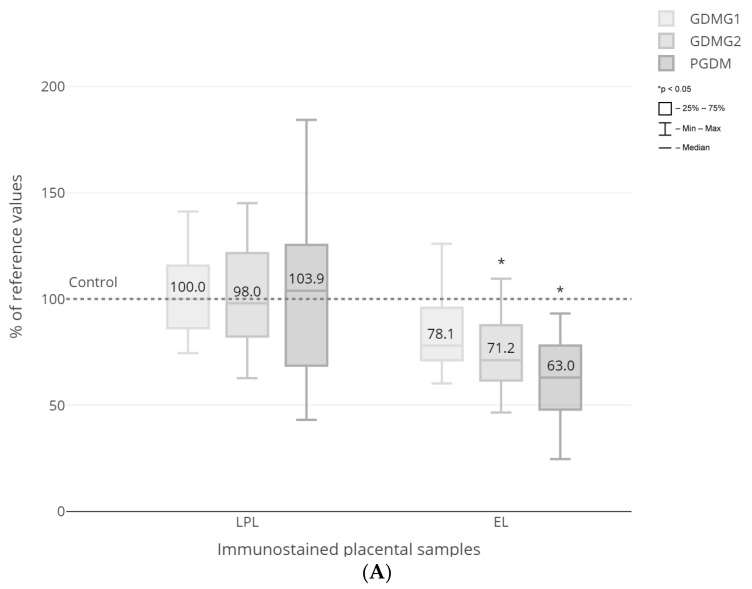

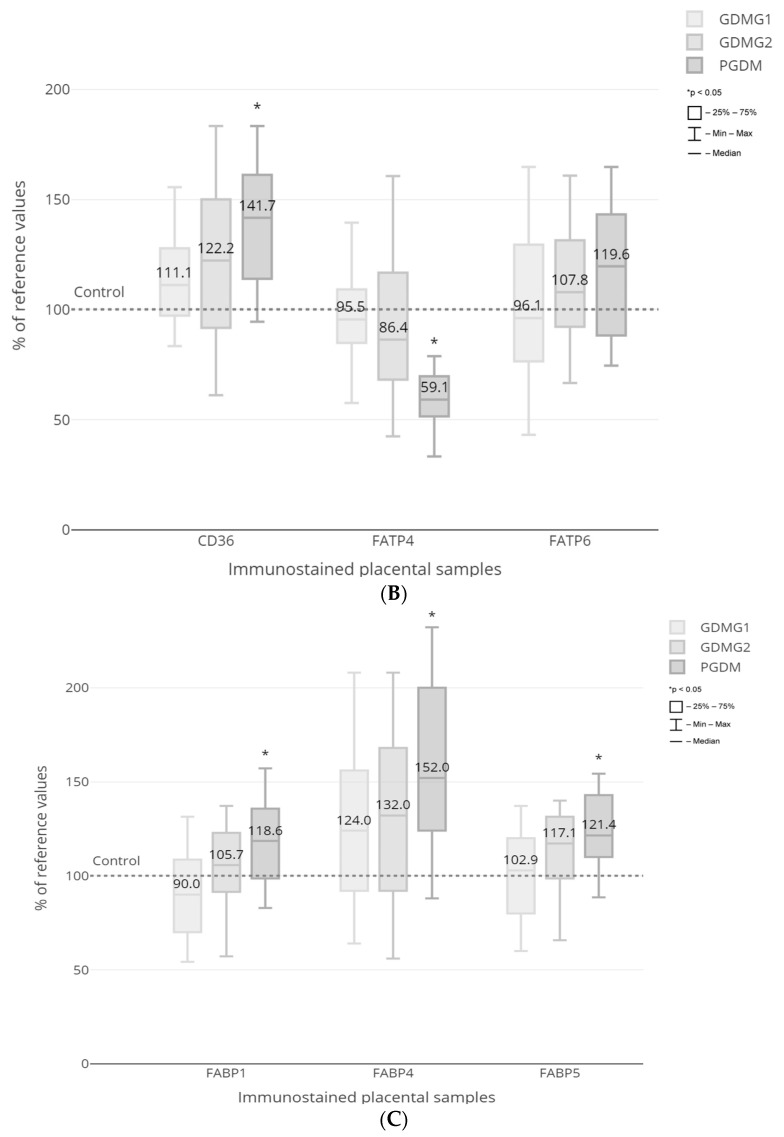
Expression of the respective proteins in placental sections: LPL, EL (**A**); CD36, FATP4, FATP6 (**B**); FABP1, FABP4, FABP5 (**C**). Results are presented as median of the percent values and IQR. The median value in the respective controls was taken as 100%. GDMG1—diet-controlled gestational diabetes mellitus; GDMG2—insulin-controlled gestational diabetes mellitus; PGDM—type 1 diabetes mellitus.

The intra- and inter-observer agreement in the digital image evaluation was assessed as substantial by the kappa analysis. Figure 3 presents all the *ĸ* values with the majority exceeding 0.61 (Figure 4). Quantitative morphometry performed for the vascular density-matched placental samples demonstrated a significant increase in the expression of CD36, FABP1, FABP4 and FABP5 proteins as well as a decrease in the expression of EL in PGDM-complicated pregnancies as compared to GDMG1 and control groups (*p* < 0.05) (Figure 5A–C). In addition, the expression of FATP4 protein was significantly decreased among patients with pre-existing type 1 diabetes as compared to both GDM and control groups (*p* < 0.05) (Figure 5B). Finally, the expression of the EL was significantly lower among women with GDM treated with insulin when compared to healthy control patients (*p* < 0.05) (Figure 5A). No significant differences with regard to the placental expression of LPL and FATP6 proteins between GDM/PGDM and non-diabetic women were noted (Figure 5A,B).

Correlation analysis revealed the presence of twelve significant associations between the expression of lipid transporters and selected maternal–fetal parameters. Among patients with GDMG1, a correlation was found between the expression of EL and FBW as well as FABP5 and fasting plasma glucose. The former was moderately negative (rho = −0.50, *p* < 0.05), whereas the latter was strong and positive (rho = 0.66, *p* < 0.001). In patients with GDM treated with insulin, CD36 protein expression remained at moderately positive correlation with pre-pregnancy weight (rho = 0.49, *p* < 0.05) and BMI (rho = 0.49, *p* < 0.05). In the same group of women, FABP4 was strongly and positively associated with pre-pregnancy weight (rho = 0.58, *p* < 0.01) and BMI (rho = 0.64, *p* < 0.01), whereas it was moderately negative with gestational weight gain (rho = −0.49, *p* < 0.05). Among patients with PGDM, a strong, positive correlation was observed between the expression of EL and pre-pregnancy weight (rho = 0.51, *p* < 0.05), as well as a moderately positive association between the FABP4 and gestational weight gain (rho = 0.49, *p* < 0.05). In the control group, there were three moderately negative correlations, i.e., between the FABP5 and pre-pregnancy weight (rho = −0.47, *p* < 0.05), FABP5 and BMI (rho = −0.48, *p* < 0.05) as well as between the FATP6 expression and fasting plasma glucose (rho = −0.50, *p* < 0.05). No statistically significant correlations between the LPL, FATP4 and FABP1 expression and selected maternal–fetal parameters in any of the patient subgroups were noted.

The results of multivariate regression analysis demonstrating independent contributors to the FBW are shown in Table 2. In the constructed model, maternal pre-pregnancy weight and BMI, placental weight as well as the expression of LPL and FABP4 proteins were selected as the strongest predictors of FBW.

## 3. Discussion

In the present study, we evaluated the expression of proteins involved in the transplacental transfer of free fatty acids in term pregnancies complicated by gestational and type 1 diabetes mellitus. The main findings of this study are threefold. Firstly, we found significant alterations in the placental expression of several lipid-transporting proteins, including EL, CD36, FATP4, FABP1, FABP4 and FABP5. Secondly, the majority of alterations occurred among women with PGDM who delivered significantly heavier neonates. Lastly, two of the analyzed proteins—LPL and FABP4—were selected as independent contributors to the FBW.

The hydrolysis of TG into FFA constitutes the first step in the cascade of processes occurring in the placental trophoblast, hence it is of critical importance for maternal–fetal lipid transfer. In concordance with previous studies, we confirmed the presence of both lipases—LPL and EL—in the trophoblast and vascular endothelium of the human term placenta [11,17,22,23,25]. Results of the morphometric analysis demonstrated no differences in the expression of LPL between diabetic and normoglycemic pregnancies. In contrast, the expression of EL was significantly lower among GDMG2/PGDM women. With respect to the former enzyme, similar results were obtained by other authors [15,22,23]. Magnusson et al. observed, however, an increase in the activity of the LPL by 39% in placentas derived from PGDM-complicated pregnancies, and hypothesized that the discrepancy between expression and activity may indicate a certain form of regulation, including LPL DNA methylation or protein dimerization [15,25,26]. Interestingly, enhanced enzyme activity was observed also among women with GDM [27]. Confirmation of the functional superiority of LPL activity over expression may be provided by the results of our multivariate regression analysis, which indicated LPL as a significant contributor to the FBW, even though no differences in the protein levels were detected. In support of these findings, in a recently published study, LPL activity rather than gene expression was positively correlated with the neonatal adiposity [28]. According to the in vitro studies, factors such as increased estradiol concentration or the combined effect of hyperglycemia and hyperinsulinemia may be responsible for the increase in LPL activity [29]. Both conditions occur in PGDM women, either due to natural or iatrogenic causes [30,31]. Consistent with the above-mentioned data, the majority of studies evaluating LPL mRNA revealed no alterations in placentas derived from GDM/PGDM-complicated pregnancies [17,18,22,32]. Nonetheless, in some recent publications evaluating placental LPL in populations of GDM women treated with diet and/or insulin, protein expression was increased and remained in positive correlation with the FBW and placental TG content, or, in complete contrast, was significantly lower when compared to normoglycemic patients [11,20,24].

In two previous studies, placental EL mRNA remained unaltered or significantly increased among lean-GDM/well-controlled PGDM or obese-GDM/poorly-controlled PGDM women, respectively [16,17]. In contrast, Barrett et al. demonstrated no changes in the EL mRNA and protein expression in placentas derived from obese GDM patients with optimal glycemic control, whereas others noted a significant decrease in the EL mRNA expression among lean-GDM women [18,22,32]. Finally, in the studies by Ruiz-Palacios et al. and Hulme et al. evaluating EL protein expression in placentas of GDMG1/GDMG2 and PGDM women, the authors were unable to demonstrate any significant differences [20,23]. The results of our study partially corroborate the above-mentioned data as we found no differences in EL expression between lean patients with GDM treated with diet and non-diabetic controls, although a tendency to decrease was observed. Surprisingly, among women with GDMG2 and PGDM, protein expression was significantly lower, even taking into account optimal glycemic control and higher BMI in both groups of patients. The above discrepancies may be explained by the fact that, as in the case of LPL, differences between EL mRNA and protein expression may result from post-transcriptional modification. Moreover, in previous studies, whole placental homogenates including enzyme-rich endothelial cells were used, whereas in the current report, we primarily focused on the trophoblast tissue [16,17,20,22]. Notably, in the study by Gauster et al., the authors observed the suppressive effect of leptin and TNF-α on the EL mRNA expression in trophoblast cells, and it is documented that concentrations of both cytokines are significantly elevated in the maternal serum in pregnancies complicated by GDM/PGDM [16,33,34,35].

The available studies on the fatty acid transport proteins in placentas derived from GDM-complicated pregnancies failed to produce unambiguous results. In a population of GDM women, Segura et al. and Zhou et al. observed significantly higher CD36 and FATP6 mRNA and protein expression in conjunction with a decreased FATP4 level [18,24]. In contrast, two recent reports demonstrated no significant changes with regard to CD36 and FATP4 mRNA or protein expression among GDMG1 and GDMG2 women [20,32]. Our morphometric findings support the latter observations. At the same time, a significant increase in CD36 and decrease in FATP4 protein expression among PGDM women was noted. It has been experimentally proved that the overexpression of CD36 and FATP4 increases the FFA uptake in rat skeletal muscle, while insulin is a well-known stimulant of both proteins trafficking towards sarcolemma [36,37]. The importance of the reduced expression of FATP4 in our population of patients with PGDM remains to be elucidated; it may be a kind of protective mechanism against excessive FFA uptake.

Similarly to FATPs, in the majority of studies evaluating the placental expression of FABP1 and FABP4 among GDM women, the authors did not observe any significant alterations [18,20,32]. Contrarily, in pregnancies complicated by the PGDM expression of FABP1 was markedly elevated [15]. In accordance with the above-mentioned data, our results revealed a significant increase in the expression of FABP1, FABP4 and FABP5 in pregnancies with concomitant PGDM and no changes in the GDM group. Furthermore, FABP4 was selected as an independent contributor to the FBW. Interestingly, in the study by Ruiz-Palacios et al., the authors observed positive correlations between the FABP4 and phosphorylated protein kinase B (p-Akt) and phosphorylated extracellular signal regulated kinase (p-ERK) expression, as well as demonstrating a significant increase in FABP4 expression following incubation with insulin in BeWo choriocarcinoma cells [20]. It could thus be speculated that FABP4 represents an insulin-responsive transporter isoform in the placenta. To further support this hypothesis, FABP4 and FABP5 mRNA and protein levels are significantly higher among obese women with type 2 diabetes mellitus, while a combination of insulin and fatty acids enhances the expression of FABP4 protein in cultured trophoblast cells alongside increased lipid droplet accumulation [19].

The majority of alterations observed in the present study occurred in pregnancies affected by PGDM. Of importance, in the same group of patients, both the FBW and the percentage of fetuses weighing ≥4000 g were the highest. As already mentioned, type 1 diabetes mellitus is associated with increased LPL activity as well as high placental TG content increasing with the maternal serum HbA1c level [15,17]. Our computed morphometric analysis revealed the increased expression of several proteins facilitating the uptake of FFA, including CD36, FABP1, FABP4 and FABP5. Hence, we can speculate that the observed changes in the transporter expression could contribute to the enhanced lipid availability for the fetus, and as a result indirectly lead to fetal overgrowth among PGDM women.

The limitations of the current study include the lack of simultaneous determination of the lipid profile in maternal serum and neonatal cord blood, which would enable a more detailed analysis of the interactions between the maternal–fetal metabolism and placental transporter expression. Furthermore, the activity of the two enzymes—LPL and EL—was not evaluated. As described in previous studies, there is an apparent divergence between LPL protein expression and activity in the human placenta [15]. Finally, the heterogeneity of GDM phenotypes should be emphasized, as well as the role of placental inflammatory response in the pathogenesis of diabetic complications. In the present study, we did not analyze the concentrations of cytokines in the maternal serum and placental tissue, and it was suggested that low-grade, metabolically induced inflammation in women with GDM may influence fetal development by impacting placental function [38]. This should be addressed in future reports. Nonetheless, the current study assessed the expression of several major proteins facilitating the transfer of FFA across the placenta in a relatively large, properly selected and well-controlled group of women diagnosed with GDM/PGDM.

In conclusion, the results of the present study revealed significant changes in the expression of proteins involved in the transfer of free fatty acids in the human diabetic placenta at term. The fact that most of the observed alterations occurred in pregnancies complicated by PGDM, characterized by the highest fetal birth weight and the percentage of macrosomic fetuses, indicates the possible impact of disturbances in the transplacental transfer of lipids on the intensification of intrauterine fetal growth in women with pre-existing diabetes mellitus.

## 4. Material and Methods

### 4.1. Reagents

Rabbit monoclonal recombinant antibody to LPL (MA5-32688), rabbit polyclonal antibody to CD36 (PA5-27236), rabbit polyclonal antibody to FATP4 (PA5-82293), rabbit polyclonal antibody to FATP6 (PA5-107262), rabbit polyclonal antibody to FABP4 (PA5-30591), rabbit polyclonal antibody to FABP5 (PA5-79232) and horse radish peroxidase (HRP) conjugated secondary goat anti-rabbit antibody (#31460) were obtained from Invitrogen, Waltham, MA, USA. Rabbit polyclonal antibody to EL (BS-10188R) was purchased from Bioss Antibodies, Woburn, MA, USA. Rabbit monoclonal antibody to FABP1 (ab171739) and rabbit polyclonal antibody to CD31 (ab28364) were obtained from Abcam, Waltham, MA, USA. IHC Select^®^ HRP/DAB kit was purchased from Merck Millipore, Darmstadt, Germany.

### 4.2. Patients

Eighty women, who delivered in the period from January to December 2022, at the 1st Department of Obstetrics and Gynecology, Medical University of Warsaw, were enrolled for the study. Participants were recruited continuously from the outpatient ambulatory dedicated for pregnancies complicated by GDM/PGDM to ensure a uniform diagnostic and therapeutic process. The study protocol was approved by the Local Ethics Committee (reference no. KB/150/2013) and all subjects signed a written informed consent before performing any procedures. The inclusion criteria were as follows: patient’s age ≥ 18 years, singleton pregnancy and gestational age > 37 weeks. Pregnancies complicated by fetal malformations, intrauterine fetal growth restriction, pre-eclampsia, maternal chronic or pregnancy-induced hypertension, chronic renal or hepatic disease, as well as those conceived by assisted reproductive technologies were excluded from the study. Tobacco use during gestation constituted an additional exclusion criterion.

The diagnosis of GDM was based on 75 g oral glucose tolerance test (OGTT) performed between 24 and 28 gestational weeks, and according to the criteria defined by the World Health Organization [39]. GDM patients received dietary advice at the initial stage of therapy and insulin was introduced only in the case of inadequate glycemic control (fasting blood glucose level >90 mg/dL and/or 1 h postprandial blood glucose level > 140 mg/dL). Women with pre-existing diabetes mellitus received insulin over the entire course of the pregnancy. To assess glycemic control in all subjects with GDM/PGDM, blood glycosylated hemoglobin (HbA1c) concentration was analyzed prior to delivery using an immunoturbidimetric method (VITROS 5600, Ortho Clinical Diagnostics, Raritan, NJ, USA; coefficient of variation < 2%; normal range: ≤6%).

### 4.3. Ultrasound

In order to assess the utero–placental and umbilical blood flows in each participant, an ultrasound examination was performed within the 72 h period prior to the vaginal/cesarean delivery, using a Voluson E8 ultrasound device (GE Healthcare, Chicago, IL, USA) equipped with 1–5 MHz convex transducer, by one experienced operator (PJS). Measurement of the umbilical artery pulsatility index (UA PI) was performed within a free-floating loop and uterine artery pulsatility index (UtA PI) was assessed below the crossing with external iliac artery. Following identification of the vessels using color mapping, a sample gate of 2 mm was placed in the central portion of the vessel and pulsed Doppler was activated. After correction of the insonation angle (<30°), tracings of at least five waves were obtained, and PI was measured automatically. With regard to UtA, both right and left vessels were scanned and mean PI was calculated.

### 4.4. Immunohistochemistry

Immediately following delivery, two separate cross-section specimens from the central and peripheral regions of the placenta were obtained from each study participant, fixed in a 10% buffered formalin, embedded in paraffin and cut into 5 μm thick sections. In total, twenty four sections (three for each transporting protein) were prepared from each of the placental specimens. Immunohistochemical staining was performed using an IHC Select^®^ HRP/DAB kit according to the protocol recommended by the manufacturer. Briefly, the paraffin was removed with xylene and sections were rehydrated with a graded series of ethanol washes before treatment with 10 mM citrate buffer (pH 6.0) for 10 min. Endogenous peroxidase was inactivated following incubation of sections with 3% H_2_O_2_ for 20 min. Sections were subsequently submerged in a blocking buffer (10% normal goat serum with 1% bovine serum albumin in Tris-buffered saline, TBS) for 2 h, followed by overnight incubation with primary antibodies at 4° C (LPL, dilution 1:200; EL, dilution 1:500; CD36, dilution 1:500; FATP4, dilution 1:200; FATP6, dilution 1:500; FABP1, 1:200; FABP4, dilution 1:500; FABP5, dilution 1:500). Incubation with HRP-conjugated secondary goat anti-rabbit antibody (0.5% *v*/*v*) was performed for 1 h at room temperature and 3,3′-diaminobenzidine served as the chromogen for HRP. Finally, after counterstaining with hematoxylin, sections were dehydrated through a series of alcohol solutions and mounted. The respective negative controls were prepared simultaneously by replacing primary antibodies with rabbit pre-immune IgG diluted with TBS at the same protein concentration as that used for primary incubation. A Leica DMLB light microscope (Leica Microsystems Cambridge, Cambridge, UK) was used for capturing and examining the digital images of the immunostained placental sections.

### 4.5. Density of Placental Microvessels

All morphometric procedures were performed according to a previously published protocol [40]. Briefly, given the fact that some lipid transporters are expressed in endothelial cells (e.g., EL), it was presumed that the accuracy of morphometric analysis may be influenced by the local differences in the density of placental microvessels. To minimize any discrepancy in the results, identification of the vascular elements in the placental sections was carried out using an endothelial cell marker—rabbit polyclonal antibody against CD31 (dilution 1:50). To assess the intensity of vascularization, the vascular/extravascular tissular index (V/EVTI) was estimated in the calibrated areas of the placental sections using a light microscope equipped with quantitative morphometry software (Quantimet 500C+ Image Processing and Analysis System, Leica Cambridge Ltd., Cambridge, UK). Briefly, in each paraffin section, three randomly selected areas were analyzed. All the analyses were repeated by two independent observers and the single visual field measured with the picture analyzer amounted to 710,055 μm^2^. The image analysis procedure consisted of the measurement of the total vascular area, i.e., total lumen area of all types of the identified vessels. To minimize the risk of disruptions caused by technical errors, in particular by use of uniaxial section of the vessel, the lowest value of the Ferret’s diameter was considered as the diameter of a single lumen.

### 4.6. Morphometric Analysis of Lipid Transporter Expression

Evaluation of the expression of lipid transporting proteins in stained placental sections was performed by means of computer-assisted quantitative morphometry (Quantimet 500C+) under light microscopy. All morphometric procedures were carried out twice by two independent researchers and, for comparative purposes, only vascular density-matched samples were analyzed. A single analyzed visual field amounted to 136,655 μm^2^ (magnification 400×) and, in each case, the difference between median V/EVTI values did not exceed ±5%. Intensity of immunostaining was evaluated using mean color saturation parameters and thresholding in grey-level histograms. As a result, expression of the respective proteins corresponded to the total immunostained calibrated area of the examined sections, where color saturation comprises segmentation-separation criteria for the objects.

### 4.7. Statistical Analysis

Statistical analyses were performed using the R^3^ v. 4.0.5 (The R Foundation for Statistical Computing, Vienna, Austria). For comparisons between continuous and categorical variables, the Kruskal–Wallis rank sum test with post hoc Dunn’s test, and the chi-square test with the Bonferroni correction were applied, respectively. All data were expressed as median and interquartile range [IQR], or as frequency (%). Statistical significance was set at *p* < 0.05.

The associations between protein expression and selected maternal–fetal parameters including maternal pre-pregnancy weight and body mass index (BMI), maternal height, gestational weight gain, gestational age, glucose concentrations during OGTT, 3rd trimester HbA1c concentration as well as placental weight and FBW, were evaluated with the use of Spearman’s rank correlation coefficient (rho). A multivariate linear regression model was created in order to determine independent predictors contributing to the FBW. Explanatory variables were selected from the group of parameters included in the correlation analysis with the addition of fetal sex and group affiliation. Prior to modeling, variables related to protein expression were standardized owing to their distribution. In order to obtain the optimal model, a backward stepwise regression approach was utilized to select potential predictors, while minimizing the Akaike Information Criterion (AIC). This method follows the principle of selecting the model with the lowest AIC value from the available options. The stepwise procedure begins by evaluating the model encompassing all variables. Then, in each iteration, the least informative variable is removed, potentially increasing the AIC value. This iterative process continues until further elimination of variables results in a deterioration of the AIC.

For the assessment of the inter- and intra-observer agreement in immunohistochemical images interpretation, the kappa statistic (*ĸ*) was used as previously described [40].

## Figures and Tables

**Table 1:** Maternal and neonatal characteristics in diabetic and control populations.

	GDMG1 (*n* = 20)	GDMG2 (*n* = 20)	PGDM (*n* = 20)	Control (*n* = 20)	*p*-Value
Age (years)	31 [26–35]	34 [32–37]	31.5 [28.8–35.3]	31 [28.5–34.3]	0.15
Gestational age (weeks)	39 [38–39]	39 [38.8–39]	38 [37–38]	39 [38–40]	<0.001 ^4,5,6^
Gravidity	2 [2–3]	2 [2–2]	1.5 [1–3]	2 [1–2]	0.22
Parity	2 [1.8–2.3]	2 [1.8–2]	1 [1–2.3]	1 [1–2]	0.16
Pre-pregnancy weight (kg)	62.5 [55.5–75]	69.5 [59.8–80.8]	69.5 [60.5–75.8]	59.5 [55–66]	0.06
Gestational weight gain (kg)	8.5 [7.8–13]	11 [8.8–12]	15.5 [9.8–19.3]	13 [10.8–15]	<0.05 ^5,6^
Height (m)	1.64 [1.6–1.67]	1.66 [1.62–1.68]	1.66 [1.64–1.68]	1.65 [1.6–1.69]	0.69
Pre-pregnancy BMI (kg/m^2^)	23.2 [21.5–26.2]	25.2 [23–30.1]	25.7 [22.9–26.5]	21.4 [20.4–24.9]	<0.05 ^2,4^
Pre-pregnancy obesity (BMI ≥ 30 kg/m^2^)	2 (10%)	3 (15%)	0	0	0.12
Fasting plasma glucose (mg/dL) ^a^	91 [88–94.3)	98.8 [95.5–104.3]	-	80 [75.3–83.8]	<0.01 ^1,2,3^
1 h plasma glucose (mg/dL) ^a^	167.5 [148.5–183.3]	188.5 [139.8–208.5]	-	122 [100.5–137]	<0.001 ^1,2^
2 h plasma glucose (mg/dL) ^a^	153 [127–161]	144 [102.8–171.3]	-	104.5 [86.5–115.3]	<0.01 ^1,2^
3rd trimester HbA1c (%)	5.1 [5–5.4]	5.4 [5–5.5]	5.9 [5.6–6.1]	-	<0.001 ^5,6^
Fetal sex					0.40
Male	7 (35%)	11 (55%)	12 (60%)	9 (45%)	
Female	13 (65%)	9 (45%)	8 (40%)	11 (55%)	
Fetal birth weight (g)	3410 [3231.3–3617.5]	3340 [3085–3767.5]	3732.5 [3430–4152.5]	3425 [3097.5–3630]	<0.05 ^4,5,6^
Placental weight (g)	523 [467–679]	576.5 [505.8–673.2]	705 [618.5–719]	612.5 [497.8–646]	<0.05 ^4,5,6^
Fetal macrosomia ^b^	2 (10%)	2 (10%)	8 (40%)	3 (15%)	0.08
UA PI	0.84 [0.73–0.94]	0.82 [0.76–0.96]	0.81 [0.75–0.85]	0.84 [0.75–0.93]	0.85
UtA PI	0.86 [0.75–0.98]	0.82 [0.7–0.93]	0.87 [0.74–1]	0.82 [0.7–0.96]	0.52

Data are expressed as median [interquartile range, IQR], or as *n* (%). GDMG1, diet-controlled gestational diabetes mellitus; GDMG2—insulin-controlled gestational diabetes mellitus; PGDM—type 1 diabetes mellitus; BMI—body mass index; HbA1c—glycated hemoglobin concentration; UA PI—umbilical artery pulsatility index; UtA PI—uterine artery pulsatility index. ^a^ Results of the 75 g oral glucose tolerance test performed between 24 and 28 gestational weeks. ^b^ Fetal macrosomia defined as birth weight over 4000 g irrespective of gestational age. ^1^ Control vs. GDMG1; ^2^ control vs. GDMG2; ^3^ GDMG1 vs. GDMG2; ^4^ control vs. PGDM; ^5^ GDMG1 vs. PGDM; ^6^ GDMG2 vs. PGDM.

**Table 2:** Results of the linear multivariate regression analysis: factors contributing to fetal birth weight.

	Coefficient	95% CI	*p*-Value
LPL *	85.6	13.4–157.8	<0.05
FABP4 *	78.2	3.9–152.6	<0.05
Pre-pregnancy weight (kg)	25.4	12.4–38.4	<0.001
Pre-pregnancy BMI (kg/m^2^)	40.6	3.8–77.4	<0.05
Fetal sex—male	135.3	−5.5–276	0.06
Placental weight (g)	2.2	1.4–2.9	<0.001

Dependent variable: fetal birth weight. Explanatory variable: group affiliation (GDMG1, GDMG2, PGDM, control), gestational age, maternal pre-pregnancy weight and BMI, maternal height, gestational weight gain, glucose concentrations during OGTT, 3rd trimester HbA1c concentration, placental weight, fetal sex, lipid transporter expression (LPL, EL, CD36, FATP4, FATP6, FABP1, FABP4, FABP5). * variables standardized before modeling. GDMG1—diet-controlled gestational diabetes mellitus; GDMG2—insulin-controlled gestational diabetes mellitus; PGDM—type 1 diabetes mellitus; BMI—body mass index; OGTT—75 g oral glucose tolerance test; CI—confidence interval.

## Data Availability

The data that support the findings of this study are available from the corresponding author on reasonable request. The data are not publicly available due to privacy or ethical restrictions.
